# High-Mobility Group Box-1 and Its Potential Role in Perioperative Neurocognitive Disorders

**DOI:** 10.3390/cells10102582

**Published:** 2021-09-28

**Authors:** Sarah Saxena, Véronique Kruys, Raf De Jongh, Joseph Vamecq, Mervyn Maze

**Affiliations:** 1 Department of Anesthesia, University Hospital Center (CHU de Charleroi), 6000 Charleroi, Belgium; sarahksaxena@gmail.com; 2 ULB Immunology Research Center (UIRC), Laboratory of Molecular Biology of the Gene, Department of Molecular Biology, Free University of Brussels (ULB), 6041 Gosselies, Belgium; vkruys@ulb.ac.be; 3 Department of Anesthesia, Fondation Hopale, 62600 Berck-sur-Mer, France; raf.dejongh@skynet.be; 4 Inserm, CHU Lille, Université de Lille, CHRU Lille, Center of Biology and Pathology (CBP) Pierre-Marie Degand, EA 7364 RADEME, 59000 Lille, France; joseph.vamecq@inserm.fr; 5 Laboratory of Hormonology, Metabolism-Nutrition & Oncology (HMNO), Department of Biochemistry and Molecular Biology, University of North France, 59000 Lille, France; 6 Center for Cerebrovascular Research, Department of Anesthesia and Perioperative Care, UCSF, San Francisco, CA 94143, USA

**Keywords:** neuroinflammation, cognitive decline, HMGB1, high mobility group box 1

## Abstract

Aseptic surgical trauma provokes the release of HMGB1, which engages the innate immune response after binding to pattern-recognition receptors on circulating bone marrow-derived monocytes (BM-DM). The initial systemic inflammation, together with HMGB1, disrupts the blood–brain barrier allowing penetration of CCR2-expressing BM-DMs into the hippocampus, attracted by the chemokine MCP-1 that is upregulated by HMGB1. Within the brain parenchyma quiescent microglia are activated and, together with the translocated BM-DMs, release proinflammatory cytokines that disrupt synaptic plasticity and hence memory formation and retention, resulting in postoperative cognitive decline (PCD). Neutralizing antibodies to HMGB1 prevents the inflammatory response to trauma and PCD.

## 1. HMGB1: An Ubiquitous and Multifunctional Protein Involved in Inflammation

### 1.1. Nomenclature

HMGB1 is an abundant nuclear protein belonging to the superfamily of High Mobility Group (HMG) proteins; the appellation, “HMG”, refers to the high electrophoretic mobility of these proteins in polyacrylamide gels. HMG proteins are classified in three major subgroups, namely HMGA, HMGB and HMGN, based on their structural properties. The HMGA proteins are small, intrinsically disordered proteins that fold upon binding into the minor groove of AT-rich DNA segments. HMGBs fold into L-shaped domains that bind to DNA with limited sequence specificity, and leads to DNA bending and chromatin decompaction. HMGN proteins are associated with the nucleosome between the histone core and DNA and are composed of a nucleosome binding domain together with an acidic chromatin unfolding domain [1].

### 1.2. Structure

**Figure 1:**
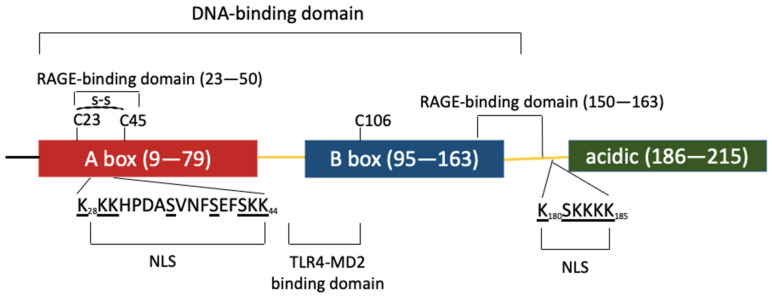
HMGB1 is composed of three domains represented by colored boxes. The A (red) and B (blue) boxes are involved in DNA binding. These DNA-binding domains are followed by an acidic C-terminal region (green). The three domains are separated by linker regions (yellow). Two nuclear localization signals (NLS1 and 2) are involved in HMGB1 nuclear import; one is located within box A and the other is positioned in the linker region before the acidic domain. The post-translationally modified amino acids within NLS1 and 2 are underlined. K and S residues are acetylated and phosphorylated, respectively. These post-translational modifications impair HMGB1 interaction with karyopherin α1, favoring HMGB1 cytoplasmic accumulation and secretion. The domains involved in HMGB1 binding to different membrane receptors are also specified.

The HMGB protein family is composed of three members (HMGB1, −2 and −3), is evolutionarily conserved from metazoans to mammals and originally arose from the fusion of intronless genes [2]. Human and rodent HMGB1 are identical except for two positions in the acidic tail and the three human HMGB proteins share ≈ 80% identity. The high degree of conservation of these proteins highlights their functional importance and implies similar biological functions. Although highly similar, *HMGB* genes differ in their expression pattern. All three *HMGB* genes are expressed during development; however, while *HMGB1* is highly expressed in all adult tissues except in brain neurons, *HMGB2* and *−3* are only active in testis and lymphoid organs at the adult stage [3]. HMGB1 has a tripartite domain organization with two HMG boxes A and B, connected by a short linker and followed by an acidic C-terminal sequence composed of 30 aspartate and glutamate residues. HMGB1 contains two lysine-rich nuclear localization signals (NLS), one located in A box and the second between B box and the acidic tail. NLS recognition by karyopherin α1 sustains HMGB1 predominant nuclear localization [4] (Figure 1).

**Figure 2:**
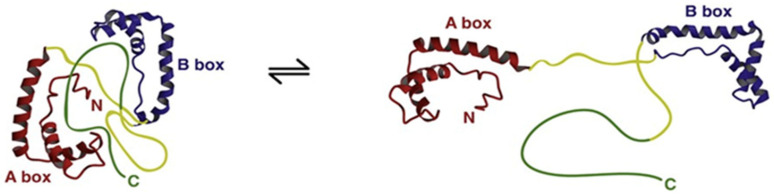
Schematic representations of a hypothetical compact, “tail-bound” form of HMGB1 in equilibrium with a more open form of the protein as deduced from dynamic structural analyses. The different domains of HMGB1 are indicated and color-coded according to Figure 1. In the “tail-bound” state, the acidic C-terminal tail is in close interaction with linkers and the concave faces of A and B boxes. In the open form, A and B boxes are available for intermolecular interactions. As the concave faces of A and B boxes are involved in HMGB1 direct interactions with DNA, the equilibrium between these two conformations probably regulates HMGB1 DNA-binding activity. Reproduced with permission from [Katherine Stott, Matthew Watson, Françoise S. Howe, J. Günter Grossmann, Jean O. Thomas], [Tail-Mediated Collapse of HMGB1 Is Dynamic and Occurs via Differential Binding of the Acidic Tail to the A and B Domains]; published by [Elsevier, 2010], [7].

Structural studies of HMGB1 demonstrated that A and B boxes of HMGB1 fold into a 3-α-helical structure to form L-shaped domains that can independently bind to the minor groove of B-type DNA, distort the double helix and induce bends of 90° or more. In vitro affinity studies revealed that HMGB1 preferentially binds non-canonical DNA structures such as single stranded DNA, as well as DNA containing cruciform or bent structures [5]. The acidic C-terminal sequence forms a flexible structure that can interact with specific positions within and between the HMG boxes and modulate DNA-binding capacities and helix-distorting activity. Moreover, these intramolecular interactions compete with HMGB1 interactions with other protein partners and regulate HMGB1 biological functions [6]. A proposed three-dimensional structural representation (Figure 2) suggests that HMGB1 exists in a dynamic equilibrium between an “open” form that can interact with DNA and a more closed form in which DNA binding domains are occluded.

### 1.3. Post-Translational Modifications

HMGB1 subcellular distribution and activity is strongly conditioned by multiple post-translational modifications. HMGB1 contains three cysteine (“C”) residues at positions 23, 45 and 106 which are sensitive to redox-dependent modifications. C23 and C45 can form a disulfide bridge and in oxidative conditions, all three cysteines can be sulfonated. The redox state of these cysteine residues strongly conditions HMGB1 functional activities [8,9].

HMGB1 is modified by several other post-translational modifications such as acetylation, phosphorylation, methylation and poly-ADP-ribosylation. The best characterized modifications include lysine acetylation and serine phosphorylation, promoting HMGB1 cytoplasmic localization and extracellular secretion [4,10].

HMGB1 can both passively and actively shuttle between the nucleus and the cytoplasm. Acetylation of lysine residues by nuclear acetyltransferases impairs HMGB1 active nuclear import, leading to redistribution of HMGB1 into the cytoplasm. This mechanism plays a major role in the cytoplasmic accumulation and subsequent secretion of HMGB1 by activated monocytes [4]. Inflammation-mediated cytoplasmic phosphorylation of serine residues within the NLS precludes HMGB1 interaction with karyopherin α1, promoting HMGB1 cytoplasmic relocalization and secretion by innate immune cells [10].

### 1.4. Extracellular Release

HMGB1 can be found in extracellular compartments. HMGB1 secretion can be due to passive release or involve active secretory mechanisms. Passive release of HMGB1 results from lytic cell death such as necrosis through rupture of the cell membrane.

Numerous studies document HMGB1 active secretion by a variety of immune and non-immune cells in response to various stimuli such as lipopolysaccharides (LPS) and proinflammatory cytokines. Devoid of a leader peptide that precludes secretion through the conventional endoplasmic reticulum–Golgi exocytic route, acetylated and/or phosphorylated cytosolic HMGB1 is actively released by the cells via exocytosis of secretory lysosomes, microparticles and exosomes. However, the signaling pathways leading to HMGB1 exocytosis, including targeting to specific vesicles, remain elusive [9]. Passively or actively released fractions of HMGB1 differ in their pattern of post-translational modifications and redox status which confer their extracellular activity [11]. While passively released HMGB1 mostly originates from the nucleus, it is non-acetylated and fully reduced. The actively secreted form is mostly acetylated and contains a C23–C45 disulfide bond [9].

### 1.5. Functions

First identified as a non-histone DNA-associated protein, HMGB1 is involved in several nuclear processes such as chromatin stabilization, replication, DNA-damage repair and gene transcription [5]. HMGB1 nuclear activities rely on the DNA-binding and -bending activities of boxes A and B and on interactions with multiple nuclear factors controlling chromatin structure and gene transcription. In normal conditions, HMGB1 is highly dynamic in the cell nucleus jumping from one chromatin site to another every second. In cells undergoing apoptosis, HMGB1 is immobilized and is stably associated with underacylated chromatin [12].

As previously mentioned, HMGB1 can reach extracellular compartments by passive release occurring upon tissue damage or can be secreted by non-canonical secretory pathways. HMGB1 was first identified as a late secreted product of LPS-activated monocytes/macrophages. As compared to the rapid and transient secretion of proinflammatory cytokines such TNF-α and IL-1β, which occurs within minutes upon exposure of monocytes/macrophages to LPS, HMGB1 is detected after several hours and remains at high levels thereafter. Similar kinetics were observed in endotoxemia mouse models. Most importantly, exposure to anti-HMGB1 prior to LPS administration in mice strongly reduces LPS-induced lethality, thereby demonstrating the permissive role of HMGB1 in endotoxemia-induced inflammation [13]. Serum HMGB1 levels are increased in septic patients as compared to healthy subjects, the highest levels were observed in non-surviving septic patients, identifying HMGB1 as a valuable clinical marker of sepsis severity/progression [13]. Since then, the inflammatory role of HMGB1 has been demonstrated in multiple infectious contexts as well as in sterile injury such as liver ischemia/reperfusion and autoimmune diseases, establishing HMGB1 as a prototypical host-derived damage-associated molecular pattern (DAMP) [14,15].

### 1.6. Molecular Signaling for Inflammation

HMGB1 is undoubtedly an important mediator of inflammation and HMGB1 neutralizing strategies have beneficial effects in preclinical models of several inflammatory diseases. To date, up to 14 different HMGB1 receptors have been described, including the receptor for advanced glycation end products (RAGE) and Toll-like receptors (TLR) which recognize both DAMPs and Pathogen-Associated Molecular Patterns (PAMPs) [15,16]. HMGB1 synergizes with several DAMPs and PAMPs to trigger postreceptor signaling and this cooperation is essential for inflammatory responses to HMGB1. HMGB1 redox status has been shown to regulate receptor binding and thereby condition the biological outcome of HMGB1 cytokine activity [17].

The best characterized HMGB1 receptors are RAGE and the LPS-binding receptor TLR4–MD2. RAGE is the first HMGB1 receptor that was identified and binds all redox forms of HMGB1. The sequence 150–183, ranging from the last 10 residues in HMGB1 box B to the start of the acidic tail, has been identified as the RAGE-binding epitope [18]. RAGE has various ligands that can trigger multiple signaling pathways by forming heterodimers with other receptors or membrane-bound signaling proteins. Whether alone or in a complex with other DAMPs such as nucleic acids, HMGB1 can activate RAGE-dependent signaling pathways leading to the nuclear relocalization of NF-kB transcription factor and, thereby, proinflammatory gene expression [19]. An immunogenic peptide located in HMGB1 box A generated by apoptosis-induced cleavage also binds to RAGE, thereby raising the possibility that HMGB1 signaling through RAGE might depend on HMGB1 interacting domain [20]. The capacity of RAGE to bind fully reduced HMGB1 accounts for the inflammatory response induced by tissue damage triggering further passive release of reduced nuclear HMGB1. Recently, it was shown that fully reduced HMGB1 promotes LPS internalization by inflammatory macrophages in a RAGE-dependent manner. Cytosolic LPS activates caspase-11 and gasdermin D-mediated pyroptosis, contributing to DAMP release and inflammation upon sepsis [21].

Interaction of HMGB1 with TLR4–MD2 receptor triggers NF-kB activation and expression of proinflammatory mediators. Binding studies revealed that while HMGB1 B box binds MD2 with low affinity and very low dissociation rate, HMGB1 A box binds TLR4 with high affinity and high dissociation rate. The capacity of HMGB1 to bind MD2 and LPS to bind TLR4 might explain the synergistic interaction between HMGB1 and LPS in triggering proinflammatory cytokine production and LPS lethality [13]. TLR4–MD2 signaling depends on the presence of C23–C25 disulfide bond and reduced C106 in HMGB1 [8,16]. Therefore, HMGB1 signaling through TLR4–MD2 activation is conditioned by the redox state of the environment. In increasing oxidative conditions, HMGB1 cysteines are fully oxidized and become irreversibly sulfonated. Fully oxidized and sulfonated HMGB1 has no cytokine activity [22].

### 1.7. Anti-Inflammatory Signaling

Growing evidence indicates a more complex role of HMGB1 in the immune response as HMGB1 is involved in mechanisms mediating immunosuppression. For example, HMGB1 contributes to anti-inflammatory mechanisms during sepsis by forming a complex with the acute phase protein haptoglobin. HMGB1-haptoglobin complex binds to CD163 receptor in macrophages and elicits the production of anti-inflammatory mediators [23]. In a tumor microenvironment, hypoxia-induced release of HMGB1 induces the accumulation of anti-inflammatory M2-like (“alternatively activated”) macrophages and IL-10 production by RAGE signaling, thereby favoring tumor development [24]. Moreover, HMGB1 induces M2-like macrophage polarization by assembling in a multimolecular signaling complex with complement component C1q, RAGE and Leukocyte-associated Ig-like Receptor 1 (LAIR-1) [25]. The HMGB1-RAGE axis also controls the adaptative immune response by promoting the migration and survival of regulatory T cells which correspond to a subset of T lymphocytes dampening T cell responses and maintaining immunological tolerance [26]. HMGB1 exerts a dampening activity on T cells by binding soluble CD52 released upon T cell activation and favoring CD54 ligation to Siglec-10 receptor, leading to the downregulation of T cell receptor signaling [27].

HMGB1 is thus a key endogenous factor controlling inflammation in response to damage or danger to the organism.

## 2. Perioperative Neurocognitive Disorders

### 2.1. Terminology

The earliest reference to cognitive problems after surgery was a short report in 1887 [28]. The first modern report of postoperative cognitive complications was by a practitioner at a geriatric hospital in England [29]. Since then, confusion has reigned not only for the suffering patients but for the investigators that were attempting to describe a constellation of neurocognitive syndromes that appeared to arise in the elderly surgical patient. Bedford’s description [29] contained terms that were descriptive (e.g., mentally inaccessible; torpid) but which did not correspond to the terminology of mental illness that existed at that time. With the advent of the cardiopulmonary bypass (CPB) technique to facilitate cardiac surgery, patients who exhibited a postoperative state of confusion, were said to be suffering from “pumphead,” in reference to the putative deleterious effect that the CPB pump had on cognitive processing. In the landmark international observational study involving non-cardiac surgery patients [30], the investigators used another non-standard term, namely, Postoperative Cognitive Dysfunction (POCD). In widespread use, POCD has proved to be a catch-all term that has no relationship to the taxonomy of neurocognitive conditions that appears in the Diagnostic and Statistical Manual of Mental Disorders (DSM).

In an attempt to categorize an increasingly disparate number of cognitive problems, Evered et al. convened The Nomenclature Consensus Working Group, with a panoply of stakeholders from the clinical and experimental community, to further refine the terminology [31]. Arising from that workshop was the all-encompassing term Perioperative Neurocognitive Disorders (PND), which included conditions that are characterized by its time of onset with respect to the surgical intervention, and to its durability and which conform to the terminology in the DSM-V. For the purposes of this review, PND will be used throughout although it is acknowledged that PND and its subdivisions do not precisely layer onto the preclinical models of these complications.

### 2.2. Healthcare Impact of Perioperative Neurocognitive Disorders (PND)

Dementia is expected to affect 75 million patients worldwide by 2030 [32]. The catastrophic impact that dementia has for the patient, their caregivers and healthcare systems cannot be overestimated [33]; however, there are no studies detailing the effect that the perioperative progressive dementia states will have globally. The additional costs of providing healthcare to patients that develop postoperative delirium in the US has been estimated to be USD 32.9 billion/year [34]. Added to that is the loss of meaningful productivity by the surgical patient and the patients’ caregivers who are both removed from their vocational lives and for whom life will likely never return to normal despite the short-lived nature of delirium [35].

### 2.3. Inflammatory Pathogenesis of Perioperative Neurocognitive Disorders

While the etiological pathways that result in PND are still not precisely known, the field has progressed since the initial reports which emphatically stated that general anesthetics were the culprit [1,29]. However, the risk of developing postoperative delirium does not differ whether the patient receives a general or a regional anesthetic [36], and in fact may be greater for those receiving regional anesthesia [37], thereby shedding doubt on the putative causal role of general anesthesia [38]. Furthermore, in cardiac surgery it was noted that the risk of developing cognitive decline postoperatively did not differ depending on whether patients received on- vs. off-pump cardiac surgery [39]; therefore, the possible deleterious effects of the “pump” as a cause of “pumphead” have also been rejected as a reason for cognitive decline in cardiac surgical patients.

Beginning with a preclinical report in which a robust hippocampal inflammatory response and a decline in hippocampal-dependent cognition were both noted in adult rats exposed to surgery [40], the possible role of aseptic trauma-induced inflammation for cognitive decline has been the focus of considerable attention. The following is a description of the results of a series of studies from a single laboratory implicating the role of HMGB1-induced inflammation for the cognitive decline that follows the aseptic trauma of surgery. In each case, rodents were exposed to aseptic surgical trauma under isoflurane general anesthesia; the trauma involved the insertion of an intramedullary pin into the tibia of the hindlimb followed by fracture of the tibia at the midpoint with osteotomy scissors.

The trauma of surgery during anesthesia in mice causes rapid release of HMGB1 into the circulation which represents a 10-fold increment over the baseline levels within one hour of the trauma [41]. That such a mechanism exists for engaging the innate immune response had previously been suggested by Matzinger in his description of the manner in which “danger” or “damage” elicits an inflammatory response that is termed a damage-associated molecular pattern or DAMP [42]. Prior to Wan et al.’s report [40], work from the laboratory of Watkins and Maier had drawn attention to hippocampal inflammation as a consequence of rats subjected to danger in the form of a brief electrical shock [43].

**Figure 3:**
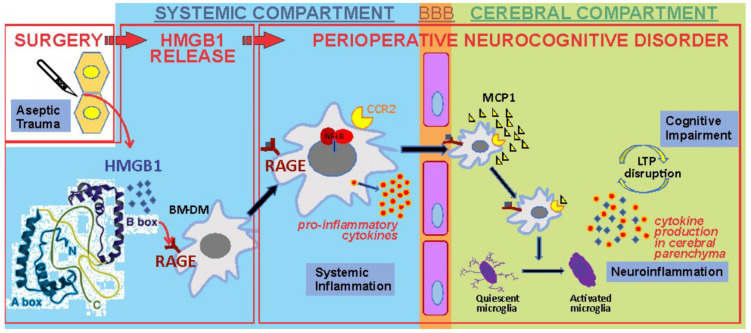
Peripheral trauma released HMGB1, which in turn engages the innate immune response by binding to receptors, including the receptor for advanced glycation end-products (RAGE), on circulating bone marrow-derived monocytes (BM-DM). This results in the translocation of the transcription factor nuclear factor kB (NF-kB) which increases the synthesis and release into the circulation of proinflammatory cytokines. These cytokines disrupt the blood–brain barrier (BBB), allowing the translocation of BM-DM into brain attracted by the chemokine monocyte chemoattractant protein-1 (MCP-1), which itself is HMGB1-dependent. In the presence of BM-DMs within the hippocampus, quiescent microglia become activated and release proinflammatory cytokines. Long-term potentiation (LTP), a synaptic plasticity mechanism required for creating and storing memory, is disrupted by the proinflammatory cytokines in the hippocampus.

Causally associated with the release of HMGB1, transcription of monocyte chemotactic protein-1 (MCP-1) rapidly increases within the hippocampus [41]. That HMGB1 is *necessary* was demonstrated with a neutralizing antibody directed against HMGB1 in which the surgery-induced neuroinflammatory cascade and behavioral changes of cognitive decline were entirely prevented [41]. That HMGB1 is itself sufficient for neuroinflammation and cognitive decline was demonstrated by fact that the disulfide form of the HMGB1 antigen produces the same inflammatory and behavioral changes as was seen after surgery [41,44]. Through HMGB1 signaling pathways in circulating bone-marrow derived monocytes (BM-DMs), NF-kB, a cytosolic transcription factor, relocates into the nucleus where it promotes the synthesis and release of proinflammatory cytokines such as, TNF-α, IL-1β and IL-6. These newly elaborated mediators of inflammation rapidly rise following trauma [45] and each of IL-1β [45], TNF-α [46] and IL-6 [47] are causally related to the downstream neuroinflammation and cognitive decline. The blood–brain barrier (BBB), which is normally an impediment to translocation of large, highly charged molecules as well as cells, becomes more permeable following trauma [48]); while both HMGB1 [49] and the proinflammatory cytokines [50] are capable of increasing BBB permeability, its roles in the mechanism of trauma-induced BBB “leakiness” has not been clearly established. MCP-1, is the chemo-attractant for the rapid influx of BM-DMs into the brain [48] where these cells become tissue macrophages working in concert with classically activated microglia [51] to further increase transcription and expression of proinflammatory cytokines and chemokines within the hippocampus. The increase in these proinflammatory mediators may be sufficient to disrupt synaptic plasticity [52] that is the basis for long-term potentiation, a neurophysiologic mechanism for learning and memory. The HMGB1-induced inflammatory response to trauma normally resolves through a combination of neural [44,48] and humoral [53] mechanisms that restores the organism to its pretrauma baseline. While these pathogenic features of trauma-induced neuroinflammation were initially demonstrated in animals, the rise in proinflammatory cytokines, including in the CSF [54], disruption of the blood–brain barrier [55], penetration of BM-DMs into the brain [56] and activation of microglia [57] have each been demonstrated in surgical patients (Figure 3).

### 2.4. Other Effects of HMGB1 on the Pathogenic Mechanisms Leading to PND

Apart from the initiation of the systemic inflammatory response that is described above, HMGB1 has also been associated with other proinflammatory actions in the pathogenic mechanism leading to neuroinflammation and cognitive decline. Recombinant HMGB1 induces morphological changes in endothelial cells and pericytes in an in vitro BBB model and, contrastingly, neutralizing antibody to HMGB1 reduced astrocyte swelling and detachment as well as opening of tight junctions using scanning electron microscopy in an in vivo ischemia–reperfusion model [58]. Similarly, in rodent models of Traumatic Brain Injury (TBI), postinjury administration of neutralizing antibody to HMGB1 prevented BBB permeability; in this circumstance RAGE receptors mediate the HMGB1-induced changes in BBB [59]. Patients with Minimal Cognitive Impairment, a predementia state, were found to have elevated circulating levels of HMGB1 that was associated with rise in soluble thrombomodulin, a marker of BBB disruption; downregulation of zona occludin-1 protein at intercellular tight junctions was deemed to be the putative reason for disruption in an in vitro model [59]. Apart from HMGB1-induced disruption of the BBB [49,58] and upregulation of the chemo-attractant MCP-1, BM-DM translocation may also be facilitated by the direct effects of HMGB1 on microvascular endothelial cells through upregulation of processes that engage rolling and flattening of circulating leukocytes [60]. HMGB1 has also been directly implicated in the activation of microglia in a model of epilepsy [61].

Consideration should also be given to the possibility that HMGB1 may be involved in the resolution of inflammation. When HMGB1 complexes with the complement protein C1q, it cross-links signaling through both RAGE and LAIR-1 changing the profile from proinflammatory to pro-resolving mediators.

## 3. Attenuation of HMGB1-Induced Neuroinflammation

Heretofore, the causal link between HMGB1 and the risk of developing PND in humans remains circumstantial. Yet, as the evidence continues to accrue in the preclinical literature, it is worthwhile to consider the therapeutic strategies that will be needed to mitigate the putative role of HMGB1 in the development of postoperative neuroinflammation and cognitive decline. Below are therapeutic strategies that can be implemented in the event that the initiating role of HMGB1 for postoperative cognitive decline is established.

### 3.1. Protein Compounds

- Monoclonal Antibody Directed at HMGB1

**Figure 4:**
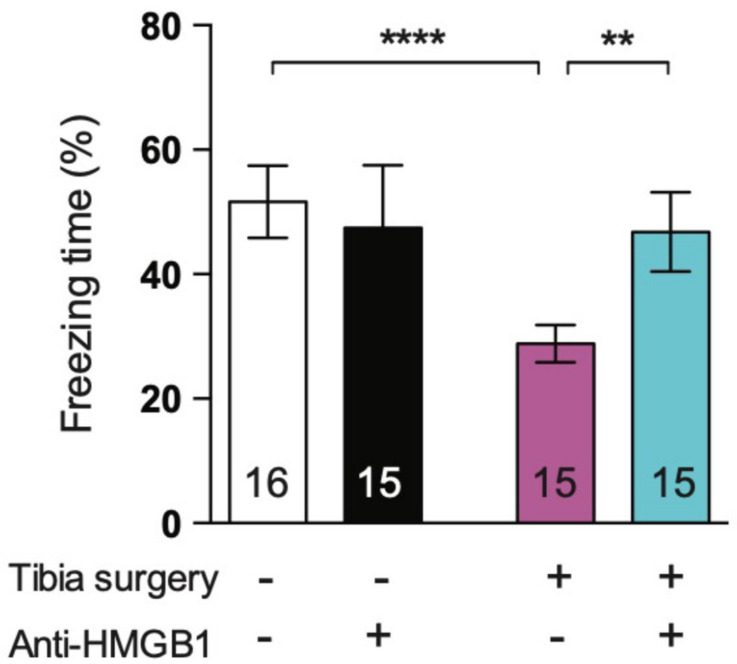
Effects of anti-HMGB1 antibody on postoperative cognitive decline. Preoperative Anti-HMGB1 antibody administration restored postoperative freezing time to control levels. (*n* = 15–16; **** *p* < 0.001 control versus surgery, and ** *p* = 0.001 surgery versus anti-HMGB1+surgery with one-way ANOVA and Bonferroni *post hoc* analysis) Reproduced with permission from [Vacas, S.; Degos, V.; Tracey, K.J.; Maze, M.], [High-mobility Group Box 1 Protein Initiates Postoperative Cognitive Decline by Engaging Bone Marrow–derived Macrophages]; published by [Wolters Kluwer Health, Inc., 2014] [41].

**Figure 5:**
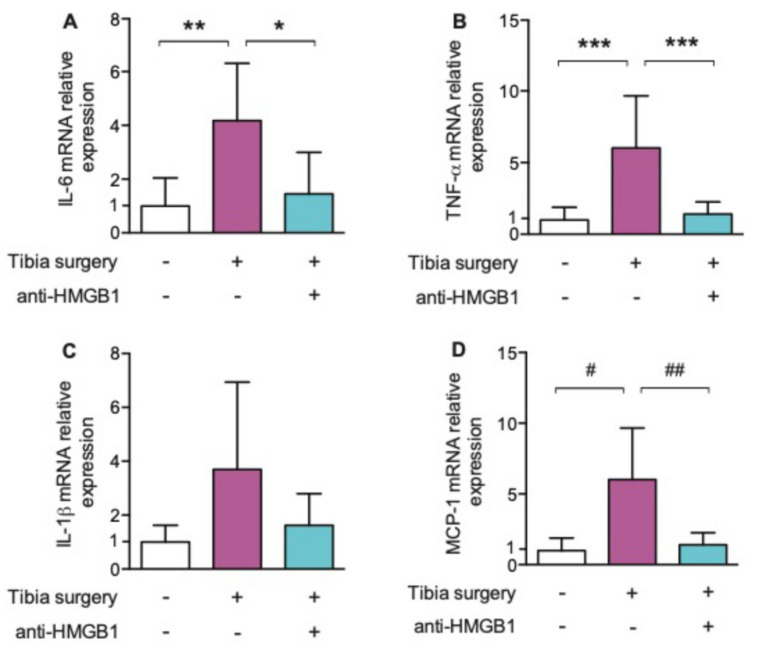
(**A**–**D**): Effects of anti-HMGB1 antibody on hippocampal interleukin (IL)-6, tumor necrosis factor (TNF)-α, IL-1β and monocyte chemotactic protein (MCP)-1 24 h postoperatively. Postoperative hippocampal Il-6; TNF- α, IL-1β and MCP-1 levels significantly decreased after anti-HMGB1 antibody (*n* = 6; * *p* = 0.023; ** *p* = 0.009; *** *p* < 0.001; # *p* = 0.003; ## *p* = 0.005 with one-way ANOVA and Bonferroni post hoc analysis) Reproduced with permission from [Vacas, S.; Degos, V.; Tracey, K.J.; Maze, M.], [High-mobility Group Box 1 Protein Initiates Postoperative Cognitive Decline by Engaging Bone Marrow–derived Macrophages]; published by [Wolters Kluwer Health, Inc., 2014] [41].

Perioperatively, in a rodent population undergoing tibia fracture surgery, HMGB1 action was blocked with the administration of neutralizing monoclonal antibody [42] This intervention led to a decrease in levels of circulating HMGB1 and IL-6 for up to 24 h postoperatively, as well as a decrease in hippocampal neuroinflammation as evidenced by a decrease in transcription of the proinflammatory IL-6 and TNF-α cytokines as well as the chemokine, MCP-1. Postoperative cognitive decline, reflected by a decrease in freezing time in the context-sensitive trace-fear conditioning paradigm, was attenuated by anti-HMGB1 (Figure 4 and Figure 5).

Similarly, the effects of neutralizing HMGB1 were also studied in rats undergoing liver surgery [62]. Anti-HMGB1 antibody was administered pre- and postoperatively and was associated with a decrease in microglial activation (measured by Iba-1 quantification in CA1 and dentate gyrus hippocampal regions) 72 h postoperatively. Liver surgery was associated with postoperative cognitive impairment (assessed in the Barnes maze paradigm); this was significantly improved by administration of the neutralizing HMGB1. Furthermore, surgical stress-induced anxiety (measured by the open field test) was also improved by negating HMGB1 function [62].

- Persistent cognitive impairment after severe sepsis remains a major challenge for survivors [63]. In a murine sepsis model involving cecal ligation and puncture (“CLP”), the elevated serum HMGB1 levels peaked 3 to 4 weeks postinjury and only returned to baseline levels by week 12; associated with these changes, the mice exhibited a significant decline in spatial memory for up to four months [64]. Administration of anti-HMGB1 antibody on days 7, 9 and 11 after the injury led to significantly reduced serum HMGB1 levels, as well as a significant improvement in spatial memory [64];
- HMGB1 release was studied in murine models of electroshock and pentylenetetrazole (PTZ)-induced convulsions. Administration of neutralizing HMGB1 was associated with a decrease in seizures in both models [65]. Anti-HMGB1 antibody administration also led to a decrease in release and translocation into the cytosol HMGB1 from the nuclei of hippocampal neurons and astrocytes. Pilocarpine induced seizures in mice were associated with BBB opening (as measured by Evans Blue leakage); exposure to the anti-HMGB1 antibody inhibited BBB leakage [66]. Pilocarpine-induced seizures are accompanied by activated microglia and astrocytes in the hippocampal CA1 region; anti-HMGB1 antibody administration attenuated glial activation [67];
- Outcome after exposure to anti-HMGB1 antibody was studied in a fluid percussion induced TBI model in mice [66]. The TBI-induced elevated circulating levels of HMGB1 and microglial activation (as evidenced by CD68 positivity) were both attenuated; functionally, TBI-induced impairment in cognition (assessed in the Morris Water Maze) and locomotion (assessed in the rotarod) were improved with anti-HMGB1 antibody [66].

B. Recombinant Box A

- Because HMGB1 binds to its receptors through Box A, strategies have been considered in which recombinant Box A is administered as a decoy preventing natural binding of the ligand to its signaling pathways on the surface of immunocytes. In an in vitro assay involving cultured macrophages, stimulation with HMGB1 to release TNF-α and IL-1β was prevented dose-dependently with recombinant Box A [68];
- In vivo experiments examining outcome in LPS-exposed mice, recombinant Box A improved survival and surviving mice were more alert and active and resumed feeding earlier [68];
- In a CLP model of sepsis, survival was improved even when the administration of recombinant Box A was administered 24 h postinjury [68].

### 3.2. Non-Protein Compounds

- Glycyrrhizin

Glycyrrhizin is a natural triterpene glycoconjugate derived from the root of licorice which binds to both A and B boxes, thereby inhibiting its chemoattractant functions in fibroblasts and smooth muscle cell [69].

- In a spinal cord ischemia–reperfusion injury (IRI) murine model circulating levels of HMGB1, IL-6, IL-1β and TNF-α were significantly lowered by glycyrrhizin [70] than in those of the control group from 2 to 72 h after reperfusion. Functionally, the degree of paraplegia in the hind-limbs was also significantly improved by glycyrrhizin [70];
- Obesity induced by a high-fat, high-fructose diet (HFHFD) in rats for 14 weeks results in systemic and neuroinflammation as well as cognitive decline. Rats that were exposed to glycyrrhizin for the final 6 weeks of the HFHFD had significant reduction in plasma and hippocampal levels of HMGB1, IL-6, IL-1β and TNF-α [71]. Similarly, diet-induced decline in spatial memory (Y maze paradigm) was prevented with glycyrrhizin [72];
- In a TBI model, rats that were treated after injury with glycyrrhizin expressed lower levels of HMGB1 in microglia/macrophages and the activated microglia changed to the M2 reparative phenotype 5 days after TBI [71]. Rotarod testing revealed a decrease in maximal speed needed to stay on the rotarod 1 day after TBI, whereas rats treated with glycyrrhizin had an improved maximal speed [71].

B. Polyunsaturated Fatty Acid (PUFA)

Specialized pro-resolving mediators (SPM) are produced from the family of *n*-3 polyunsaturated fatty acids and mediate the resolution of inflammatory responses. After TBI, rats that received PUFA exhibited a decrease in microglial activation and decreased levels of TNFα, IL-1β, IL-6, IFN-γ and HMGB1 [73].

C. Flavonoid (Baicalin)

Flavonoids, found in fruits, vegetables, chocolate and dark tea, among others, are capable of inhibiting proinflammatory transcription factors. Baicalin, a flavonoid supplement used in traditional oriental medicine, can penetrate the blood–brain barrier (BBB).

- Adult mice subjected to LPS-induced sickness behavior have glial activation (both microglia and astrocytes) with high HMGB1 levels in the hippocampal glia [74]. Exposure to baicalin prevented glial activation and elevated hippocampal proinflammatory cytokines; functionally, the LPS-induced cognitive decline (assessed in the Morris Water Maze was significantly improved [74].

## 4. Identification of Patients Prone to Enhanced Neuroinflammation/PND

Patient-related, as well as perioperative factors, can increase the risk of the development of PND. In this section we consider the putative role that HMGB1 may play in the pathogenic mechanisms that increase the risk of developing PND. The possible role that naturally circulating anti-HMGB1 IgM neutralizing antibodies may play in preventing PND [75] is of interest but is not further considered because there are, as yet, no reports describing HMGB1 antibody levels perioperatively.

### 4.1. Non-Modifiable Patient-Related Factors That Affect Development of PND

- Age

Increasing age is associated with both a higher prevalence of PND [76], as well as higher plasma levels of HMGB1 in non-surgical patients [77]. Whether the increase in age-related PND is due to higher levels of HMGB1 requires further exploration.

B. Gender

HMGB1 plasma levels are significantly higher in females [77]. There appears to be a synergistic interaction between estrogen and HMGB1 in which the levels of the former enhance the levels of the latter [78]. In addition, binding of estrogen to estrogen-responsive elements in the nucleosome is strengthened by HMGB1 [79]. In a meta-analysis of studies involving spinal surgery, females were significantly more likely to develop postoperative delirium [80]. Interestingly, other neurodegenerative conditions, in which neuroinflammation is a key pathogenic factor, are also more common in females [81]. A possible linkage between PND prevalence in females and HMGB1 has not been formally investigated.

C. Genetic and Racial Factors

Thus far the only genetic factor that appears to increase the risk of the development of PND is whether males have the Apo E4 allele [82]. There are several mutations on the HMGB1 gene that have been noted to increase the levels of circulating HMGB1 [83], however, the relevance of these mutations for the development of PND has not been established. Plasma HMGB1 levels were noted to be higher in an African American cohort compared to a Caucasian one [77], while the relative risk of developing postoperative delirium may be greater (1.3) for non-whites; the 95% confidence intervals crossed unity (0.8–2.1) [84]; the entire effect may reflect social disadvantage.

### 4.2. Pre-Existing Medical Diseases That Affect Development of PND

- Atrial fibrillation (AF)

The presence of AF at any stage of life is accompanied with an increased risk of cognitive dysfunction, but not necessarily in the perioperative period [85]. While pathological causes of cognitive decline, such as small vessel disease and neurodegeneration, are also associated with AF [86], some have speculated that HMGB1 may be responsible for eliciting atrial fibrillation [87]. Furthermore, two mutations in *HMGB1* (rs2249825^G/G^ and rs2249825^C/G^) have been found to be associated with increased risk of postoperative AF after coronary artery bypass graft surgery [88]. Additionally, the 3′UTR variant rs1045411^T/C^ of *HMGB1* may influence the risk of late-onset AF after cardiac surgery [89].

B. Obesity, diabetes mellitus and the metabolic syndrome

Plasma HMGB1 levels were higher in non-surgical individuals with higher BMI, waist circumference and waist to hip ratio [77] and HMGB1 is overexpressed in diabetic patients compared to non-diabetics [90]. Higher production of HMGB1 by adipose tissue may explain these findings [91]. Patients suffering from obesity, diabetes and the metabolic syndrome have a low-grade inflammation through activation of the innate immune system [92]. Obesity, diabetes and metabolic syndrome are risk factors for PND [93,94], although its link to higher levels of HMGB1 has not been established.

C. Obstructive Sleep Apnea (OSA)

OSA, the most common form of Sleep Disordered Breathing, is on the increase, affecting 3 to 17% of the general population depending on gender, age and body mass index [95], and substantially increases the risk of developing postoperative delirium [96], although observational studies have not detected a decline in cognitive performance [97] OSA patients have higher plasma HMGB1 levels compared to healthy controls; interestingly, HMGB1 normalized after CPAP treatment [98], an intervention that also decreases the risk of neurocognitive dysfunction [99]. The reason why OSA patients have higher HMGB1 levels may be due to the repeated bouts of hypoxemia that occur in OSA patients during sleep as simulating intermittent hypoxic conditions in mice results in higher plasma HMGB1 levels [100].

D. Gut Microbiota

A brain–gut microbiota axis involving neural, immune and endocrine pathways has been identified as a possible mechanism for increasing the risk of neuroinflammation [101], a pivotal pathogenic mechanism for PND. Given the ubiquitous use of prophylactic antibiotics in the perioperative period it is likely that the gut microbiome may be altered, which can increase the risk of prolonged postoperative neuroinflammation [102,103]. Aged mice that developed cognitive decline after aseptic trauma had an altered gut microbiome [31]; furthermore, exposure to trimethylamine N-oxide (TMAO), a gut microbiome-derived metabolite, increased the likelihood of cognitive decline following aseptic trauma [104]. Interestingly, TMAO transduces its signaling role for the development of endothelial dysfunction via HMGB1 [105,106].

E. Neurological diseases

Neurodegenerative and neurovascular diseases are associated with an increased risk of development of PND. [107] HMGB1 plays a pivotal role in the progression of these neurological conditions [108], possibly by enhancing neuroinflammation [109,110]. HMGB1 and its receptors are increased in the brains of patients with multiple sclerosis and in mice with experimental autoimmune encephalomyelitis [111], as well as in the glia of amyotrophic lateral sclerosis. In Alzheimer’s Disease, secreted HMGB1 impairs memory through pattern recognition receptors that engage the innate immune response; furthermore, the secreted HMGB1 can aggregate to neuritic plaques and then bind Aβ, which in turn inhibits the phagocytosis and degradation of Aβ by microglial cells [111].

Chronic pain states are associated with cognitive decline [112]. Interestingly, HMGB1 participates in the pathogenesis of neuropathic pain [111] and HMGB1 antibodies can diminish neuropathic pain in rats [113]. Whether increased perioperative HMGB1 can affect postoperative pain is not known.

## 5. Conclusions

This brief review has focused on the multiple functions that HMGB1 plays in the development of PND. Apart from initiating the inflammatory process that progresses to neuroinflammation that can alter learning and memory, HMGB1 may also have a role as a marker for both the risk of developing PND as well as of the progression of the disease itself. HMGB1 may represent a molecular target to disable in order to prevent the development and progression of PND; current strategies to disable HMGB1 include the use of neutralizing antibodies and post-translational modifications that may alter its functional capacity. While much remains unknown, this DAMP is likely to be a future focus of investigations into the pathogenesis and treatment of PND.

## Data Availability

Not applicable.

## References

[1] Bianchi M.E., Agresti A. (2005). HMG proteins: Dynamic players in gene regulation and differentiation. Curr. Opin. Genet. Dev..

[2] Sessa L., Bianchi M.E. (2007). The evolution of High Mobility Group Box (HMGB) chromatin proteins in multicellular animals. Gene.

[3] Müller S., Ronfani L., Bianchi M.E. (2004). Regulated expression and subcellular localization of HMGB1, a chromatin protein with a cytokine function. J. Intern. Med..

[4] Bonaldi T., Talamo F., Scaffidi P., Ferrera D., Porto A., Bachi A., Rubartelli A., Agresti A., Bianchi M.E. (2003). Monocytic cells hyperacetylate chromatin protein HMGB1 to redirect it towards secretion. EMBO J..

[5] Stros M. (2010). HMGB proteins: Interaction with DNA and chromatin. Biochem. Biophys. Acta.

[6] Knapp S., Müller S., Digilio G., Bonaldi T., Bianchi M.E., Musco G. (2004). The long acidic tail of high mobility group box 1 (HMGB1) protein forms an extended and flexible structure that interacts with specific residues within and between the HMG boxes. Biochemistry.

[7] Stott K., Watson M., Howe F.S., Grossmann J.G., Thomas J.O. (2010). Tail-mediated collapse of HMGB1 is dynamic and occurs via differential binding of the acidic tail to the A and B domains. J. Mol. Biol..

[8] Yang H., Lundbäck P., Ottosson L., Erlandsson-Harris H., Venereau E., Bianchi M.E., Al-Abed Y., Andersson U., Tracey K.J. (2021). Redox modifications of cysteine residues regulate the cytokine activity of HMGB1. Mol. Med..

[9] Deng M., Scott M.J., Fan J., Billiar T.R. (2019). Location is the key to function: HMGB1 in sepsis and trauma-induced inflammation. J. Leukoc. Biol..

[10] Youn J.H., Shin J.-S. (2006). Nucleocytoplasmic shuttling of HMGB1 is regulated by phosphorylation that redirects it toward secretion. J. Immunol..

[11] Yang H., Antoine D.J., Andersson U., Tracey K.J. (2013). The many faces of HMGB1: Molecular structure-functional activity in inflammation, apoptosis, and chemotaxis. J. Leukoc. Biol..

[12] Scaffidi P., Misteli T., Bianchi M.E. (2002). Release of chromatin protein HMGB1 by necrotic cells triggers inflammation. Nature.

[13] Wang H., Bloom O., Zhang M., Vishnubhakat J.M., Ombrellino M., Che J., Frazier A., Yang H., Ivanova S., Borovikova L. (1999). HMG-1 as a late mediator of endotoxin lethality in mice. Science.

[14] Andersson U., Tracey K.J. (2011). HMGB1 is a therapeutic target for sterile inflammation and infection. Annu. Rev. Immunol..

[15] Ge Y., Huang M., Yao Y.-M. (2021). The Effect and Regulatory Mechanism of High Mobility Group Box-1 Protein on Immune Cells in Inflammatory Diseases. Cells.

[16] Yang H., Wang H., Chavan S.S., Andersson U. (2015). High Mobility Group Box Protein 1 (HMGB1): The Prototypical Endogenous Danger Molecule. Mol. Med..

[17] Tang D., Kang R., Livesey K.M., Zeh 3rd H.J., Lotze M.T. (2011). High mobility group box 1 (HMGB1) activates an autophagic response to oxidative stress. Antioxid. Redox Signal..

[18] Huttunen H.J., Fages C., Kuja-Panula J., Ridley A.J., Rauvala H. (2002). Receptor for advanced glycation end products-binding COOH-terminal motif of amphoterin inhibits invasive migration and metastasis. Cancer Res..

[19] Kierdorf K., Fritz G. (2013). RAGE regulation and signaling in inflammation and beyond. J. Leukoc. Biol..

[20] LeBlanc P.M., Doggett T.A., Choi J., Hancock M.A., Durocher Y., Frank F., Nagar B., Ferguson T.A., Saleh M. (2014). An immunogenic peptide in the A-box of HMGB1 protein reverses apoptosis-induced tolerance through RAGE receptor. J. Biol. Chem..

[21] Deng M., Tang Y., Li W., Wang X., Zhang R., Zhang X., Zhao X., Liu J., Tang C., Liu Z. (2018). The Endotoxin Delivery Protein HMGB1 Mediates Caspase-11-Dependent Lethality in Sepsis. Immunity.

[22] Venereau E., Casalgrandi M., Schiraldi M., Antoine D.J., Cattaneo A., De Marchis F., Liu J., Antonelli A., Preti A., Raeli L. (2012). Mutually exclusive redox forms of HMGB1 promote cell recruitment or proinflammatory cytokine release. J. Exp. Med..

[23] Yang H., Wang H., Levine Y.A., Gunasekaran M.K., Wang Y., Addorisio M., Zhu S., Li W., Li J., de Kleijn D.P. (2018). Identification of CD163 as an antiinflammatory receptor for HMGB1-haptoglobin complexes. JCI Insight.

[24] Huber R., Meier B., Otsuka A., Fenini G., Satoh T., Gehrke S., Widmer D., Levesque M.P., Mangana J., Kerl K. (2016). Tumour hypoxia promotes melanoma growth and metastasis via High Mobility Group Box-1 and M2-like macrophages. Sci. Rep..

[25] Son M., Porat A., He M., Suurmond J., Santiago-Schwarz F., Andersson U., Coleman T.R., Volpe B.T., Tracey K.J., Al-Abed Y. (2016). C1q and HMGB1 reciprocally regulate human macrophage polarization. Blood.

[26] Wild C.A., Bergmann C., Fritz G., Schuler P., Hoffmann T.K., Lotfi R., Westendorf A., Brandau S., Lang S. (2012). HMGB1 conveys immunosuppressive characteristics on regulatory and conventional T cells. Int. Immunol..

[27] Bandala-Sanchez E., Bediaga N.G., Goddard-Borger E.D., Ngui K., Naselli G., Stone N.L., Neale A.M., Pearce L.A., Wardak A., Czabotar P. (2018). CD52 glycan binds the proinflammatory B box of HMGB1 to engage the Siglec-10 receptor and suppress human T cell function. Proc. Natl. Acad. Sci. USA.

[28] Savage G.H. (1887). Insanity following the use of anaesthetics in operations. BMJ.

[29] Bedford P.D. (1955). Adverse cerebral effects of anaesthesia on old people. Lancet.

[30] Moller J.T., Cluitmans P., Rasmussen L.S., Houx P., Rasmussen H., Canet J., Rabbitt P., Jolles J., Larsen K., Hanning C.D. (1998). Long-term postoperative cognitive dysfunction in the elderly ISPOCD1 study. ISPOCD investigators. International Study of Post-Operative Cognitive Dysfunction. Lancet.

[31] Everd L., Silbert B., Knopman D.S., Scott D.A., DeKosky S.T., Rasmussen L.S., Oh E.S., Crosby G., Berger M., Eckenhoff R.G. (2018). Nomenclature Consensus Working Group. Recommendations for the Nomenclature of Cognitive Change Associated with Anaesthesia and Surgery-2018. Anesthesiology.

[32] Ferri C.P., Jacob K.S. (2017). Dementia in low-income and middle-income countries: Different realities mandate tailored solutions. PLoS Med..

[33] Yemm H., Robinson D.L., Paddick S.M., Dotchin C., Goodson M.L., Narytnyk A., Poole M., Mc Ardle R. (2021). Instrumental Activities of Daily Living Scales to Detect Cognitive Impairment and Dementia in Low- and Middle-Income Countries: A Systematic Review. J. Alzheimers. Dis..

[34] Gou R.Y., Hsiej T.T., Marcantonio E.R., Cooper Z., Jones R.N., Travison T.G., Fong T.G., Abdeen A., Lange J., Earp B. (2021). One-Year Medicare Costs Associated with Delirium in Older Patients Undergoing Major Elective Surgery. JAMA Surg..

[35] Saczynski J.S., Marcantonio E.R., Quach L., Fong T.G., Gross A., Inouye S.K., Jones R.N. (2012). Cognitive trajectories after postoperative delirium. NEJM.

[36] Davis N., Lee M., Lin A.Y., Lynch L., Monteleone M., Falzon L., Ispahany N., Lei S. (2014). Postoperative cognitive function following general versus regional anesthesia: A systematic review. J. Neurosurg. Anesthesiol..

[37] Wu J., Yin Y., Jin M., Li B. (2021). The risk factors for postoperative delirium in adult patients after hip fracture surgery: A systematic review and meta-analysis. Int. J. Geriatr. Psychiatry.

[38] Evered L., Scott D.A., Silbert B., Maruff P. (2011). Postoperative cognitive dysfunction is independent of type of surgery and anesthetic. Anesth. Analg..

[39] Shaefi S., Mittel A., Loberman D., Ramakrishna H. (2019). Off-Pump Versus On-Pump Coronary Artery Bypass Grafting-A Systematic Review and Analysis of Clinical Outcomes. J. Cardiothorac. Vasc. Anesth..

[40] Wan Y., Xu J., Ma D., Zeng Y., Cibelli M., Maze M. (2007). Postoperative impairment of cognitive function in rats: A possible role for cytokine-mediated inflammation in the hippocampus. Anesthesiology.

[41] Vacas S., Degos V., Tracey K.J., Maze M. (2014). High-mobility group box 1 protein initiates postoperative cognitive decline by engaging bone marrow-derived macrophages. Anesthesiology.

[42] Matzinger P. (2002). The danger model: A renewed sense of self. Science.

[43] O’ Connor K.A., Johnson J.D., Hansen M.K., Wieseler Frank J.L., Maksimova E., Watkins L.R., Maier S.F. (2003). Peripheral and central proinflammatory cytokine response to a severe acute stressor. Brain Res..

[44] Hu J., Vacas S., Feng X., Lutrin D., Uchida Y., Lai I.K., Maze M. (2018). Dexmedetomidine Prevents Cognitive Decline by Enhancing Resolution of High Mobility Group Box 1 Protein-induced Inflammation through a Vagomimetic Action in Mice. Anesthesiology.

[45] Cibelli M., Fidalgo A.R., Terrando N., Ma D., Monaco C., Feldmann M., Takata M., Lever I.J., Nanchahal J., Fanselow M.S. (2010). Role of interleukin-1beta in postoperative cognitive dysfunction. Ann. Neurol..

[46] Terrando N., Monaco C., Ma D., Foxwell B.M.J., Feldmann M., Maze M. (2010). Tumor necrosis factor-alpha triggers a cytokine cascade yielding postoperative cognitive decline. Proc. Natl. Acad. Sci. USA.

[47] Maze M. (2018). Interleukin-6 is both necessary and sufficient to produce perioperative neurocognitive disorder in mice. Br. J. Anaesth..

[48] Terrando N., Eriksson L.I., Ryu J.K., Yang T., Monaco C., Feldmann M., Fagerlund M.J., Charo I.F., Akassoglou K., Maze M. (2011). Resolving postoperative neuroinflammation and cognitive decline. Ann. Neurol..

[49] Festoff B.W., Sajja R.K., Van Dreden P., Cucullo L. (2016). HMGB1 and thrombin mediate the blood-brain barrier dysfunction acting as biomarkers of neuroinflammation and progression to neurodegeneration in Alzheimer’s disease. J. Neuroinflamm..

[50] Lu Y., Xu Z., Shen F., Lin R., Li H., Lv X., Liu Z. (2020). Propofol Protects Against TNF-α-induced Blood-brain Barrier Disruption via the PIM-1/eNOS/NO Pathway. Curr. Neurovasc. Res..

[51] Feng X., Valdearcos M., Uchida Y., Lutrin D., Maze M., Koliwad S.K. (2017). Microglia mediate postoperative hippocampal inflammation and cognitive decline in mice. JCI Insight.

[52] Kelly A., Lynch A., Nolan Y., Queenan P., Whittaker E., O’Neill L.A., Lynch M.A. (2001). The anti-inflammatory cytokine, interleukin (IL)-10, blocks the inhibitory effect of IL-1 beta on long term potentiation. A role for JNK. J. Biol. Chem..

[53] Yang T., Xu G., Newton P.T., Chagin A.S., Mkrtchian S., Carlstrom M., Zhang X.-M., Harris R.A., Cooter M., Berger M. (2019). Maresin 1 attenuates neuroinflammation in a mouse model of perioperative neurocognitive disorders. Br. J. Anaesth..

[54] Hirsch J., Vacas S., Terrando N., Yuan M., Sands L.P., Kramer J., Bozic K., Maze M., Leung J.M. (2016). Perioperative cerebrospinal fluid and plasma inflammatory markers after orthopedic surgery. J. Neuroinflamm..

[55] Danielson M., Reinsfelt B., Westerlind A., Zetterberg H., Blennow K., Ricksten S.-E. (2018). Effects of methylprednisolone on blood-brain barrier and cerebral inflammation in cardiac surgery-a randomized trial. J. Neuroinflamm..

[56] Berger M., Oyeyemi D., Olurinde M.O., Whitson H.E., Weinhold K.J., Woldorff M.G., Lipsitz L.A., Moretti E., Giattino G.M., Roberts K.C. (2019). The INTUIT Study: Investigating Neuroinflammation Underlying Postoperative Cognitive Dysfunction. J. Am. Geriatr. Soc..

[57] Forsberg A., Cervenka S., Fagerlund M.J., Rasmussen L.S., Zetterberg H., Harris H.E., Stridh P., Christensson E., Granstrom A., Schening A. (2017). The immune response of the human brain to abdominal surgery. Ann. Neurol..

[58] Zhang J., Takahashi H.K., Liu K., Wake H., Liu R., Maruo T., Date I., Yoshino T., Ohtsuka A., Mori S. (2011). Anti-high mobility group box-1 monoclonal antibody protects the blood-brain barrier from ischemia-induced disruption in rats. Stroke.

[59] Okuma Y., Liu K., Wake H., Zhang J., Maruo T., Date I., Yoshino T., Ohtsuka A., Otani N., Tomura S. (2012). Anti-high mobility group box-1 antibody therapy for traumatic brain injury. Ann. Neurol..

[60] Fiuza C., Bustin M., Talwar S., Tropea M., Gerstenberger E., Shelhamer J.H., Suffredini A.F. (2003). Inflammation-promoting activity of HMGB1 on human microvascular endothelial cells. Blood.

[61] Shi Y., Zhang L., Teng J., Miao W. (2018). HMGB1 mediates microglia activation via the TLR4/NF-κB pathway in coriaria lactone induced epilepsy. Mol. Med. Rep..

[62] Terrando N., Yang T., Wang X., Fang J., Cao M., Andersson U., Erlandsson H.H., Ouyang W., Tong J. (2016). Systemic HMGB1 Neutralization Prevents Postoperative Neurocognitive Dysfunction in Aged Rats. Front. Immunol..

[63] Rengel K.F., Hayhurst C.J., Pandharipande P.P., Hughes C.G. (2019). Long-term Cognitive and Functional Impairments after Critical Illness. Anesth. Analg..

[64] Chavan S.S., Huerta P.T., Robbiati S., Valdes-Ferrer S.I., Ochani M., Dancho M., Frankfurt M., Volpe B.T., Tracey K.J., Diamond B. (2012). HMGB1 mediates cognitive impairment in sepsis survivors. Mol. Med..

[65] Zhao J., Wang Y., Xu C., Liu K., Wang Y., Chen L., Wu X., Gao F., Guo Y., Zhu J. (2017). Therapeutic potential of an anti-high mobility group box-1 monoclonal antibody in epilepsy. Brain Behav. Immun..

[66] Okuma Y., Wake H., Teshigawara K., Takahashi Y., Hishikawa T., Mori S., Takahashi H.K., Date I., Nishibori M. (2019). Anti-High Mobility Group Box 1 Antibody Therapy May Prevent Cognitive Dysfunction After Traumatic Brain Injury. World Neurosurg..

[67] Fu L., Liu K., Wake H., Teshigawara K., Yoshino T., Takahashi H., Mori S., Nishibori M. (2017). Therapeutic effects of anti-HMGB1 monoclonal antibody on pilocarpine-induced status epilepticus in mice. Sci. Rep..

[68] Yang H., Ochani M., Li J., Qiang X., Tanovic M., Harris H.E., Susarla S.M., Ulloa L., Wang H., DiRaimo R. (2004). Reversing established sepsis with antagonists of endogenous high-mobility group box 1. Proc. Natl. Acad. Sci. USA.

[69] Mollica L., De Marchis F., Spitaleri A., Dallacosta C., Pennacchini D., Zamai M., Agresti A., Trisciuoglio L., Musco G., Bianchi M.E. (2007). Glycyrrhizin binds to high-mobility group box 1 protein and inhibits its cytokine activities. Chem. Biol..

[70] Gong G., Yuan L., Hu L., Wu W., Yin L., Hou J.L., Liu Y.H., Zhou L.S. (2012). Glycyrrhizin attenuates rat ischemic spinal cord injury by suppressing inflammatory cytokines and HMGB1. Acta. Pharmacol. Sin..

[71] Gao T., Chen Z., Chen H., Yuan H., Wang Y., Peng X., Wei C., Yang J., Xu C. (2018). Inhibition of HMGB1 mediates neuroprotection of traumatic brain injury by modulating the microglia/macrophage polarization. Biochem. Biophys. Res. Commun..

[72] Yu M., Huang H., Dong S., Sha H., Wei W., Liu C. (2019). High mobility group box-1 mediates hippocampal inflammation and contributes to cognitive deficits in high-fat high-fructose diet-induced obese rats. Brain Behav. Immun..

[73] Chen X., Wu S., Chen C., Xie B., Fang Z., Hu W., Chen J., Fu H., He H. (2017). Omega-3 polyunsaturated fatty acid supplementation attenuates microglial-induced inflammation by inhibiting the HMGB1/TLR4/NF-κB pathway following experimental traumatic brain injury. J. Neuroinflamm..

[74] Li Y., Lui T., Li Y., Han D., Hong J., Yang N., He J., Peng R., Mi X., Kuang C. (2020). Baicalin Ameliorates Cognitive Impairment and Protects Microglia from LPS-Induced Neuroinflammation via the SIRT1/HMGB1 Pathway. Oxid. Med. Cell Longev..

[75] Geng Y., Munirathinam G., Palani S., Ross J.E., Wang B., Chen A., Zheng G. (2020). HMGB1-Neutralizing IgM Antibody Is a Normal Component of Blood Plasma. J. Immunolog..

[76] Newman S., Stygall J., Hirani S., Shaefi S., Maze M., Warltier D.C. (2007). Postoperative Cognitive Dysfunction after Noncardiac Surgery. Anesthesiology.

[77] Chen L., Zhu H., Su S., Harshfield G., Sullivan J., Webb C., Blumenthal J.A., Wang X., Huang Y., Treiber F.A. (2020). High-Mobility Group Box-1 Is Associated with Obesity, Inflammation, and Subclinical Cardiovascular Risk among Young Adults a Longitudinal Cohort Study. Arterioscler. Thromb. Vasc. Biol..

[78] Chau K.Y., Lam H.Y.P., Lee K.L.D. (1998). Estrogen Treatment Induces Elevated Expression of HMG1 in MCF-7 Cells. Exp. Cell Res..

[79] Scovell W.M. (2016). High Mobility Group Protein 1: A Collaborator in Nucleosome Dynamics and Estrogen-Responsive Gene Expression. World J. Biol..

[80] Zhang H.J., Ma X.H., Ye J.B., Liu C.Z., Zhou Z.Y. (2020). Systematic Review and Meta-Analysis of Risk Factor for Postoperative Delirium Following Spinal Surgery. J. Orthop. Surg. Res..

[81] Crespo-Castrillo A., Arevalo M.-A. (2020). Microglial and astrocytic function in physiological and pathological conditions: Estrogenic modulation. Int. J. Mol. Sci..

[82] Schenning K.J., Murchison C.F., Mattek N.C., Kaye J.A., Quinn J.F. (2019). Sex and Genetic Differences in Postoperative Cognitive Dysfunction: A Longitudinal Cohort Analysis. Biol. Sex. Dif..

[83] Kornblit B., Munthe-Fog L., Petersen S.L., Madsen H.O., Vindeløv L., Garred P. (2007). The Genetic Variation of the Human HMGB1 Gene. Tissue Antigens.

[84] Arias F., Chen F., Fong T.G., Shiff H., Alegria M., Marcantonio E.R., Gou Y., Jones R.N., Travison T.G., Schmitt E.M. (2020). Social Disadvantage and Risk of Delirium Following Major Surgery. J. Am. Geriatr. Soc..

[85] Hui D.S., Morley J.E., Mikolajczak P.C., Lee R. (2015). Atrial Fibrillation: A Major Risk Factor for Cognitive Decline. Am. Heart J..

[86] Banerjee G., Chan E., Ambler G., Wilson D., Cipolotti L., Shakeshaft C., Cohen H., Yousry T., Habil M., Al-Shahi Salman R. (2020). Cognitive Impairment Before Atrial Fibrillation-Related Ischemic Events: Neuroimaging and Prognostic Associations. J. Am. Heart Assoc..

[87] Hu X.-R., Wang X.H., Liu H.F., Zhou W.J., Jiang H. (2012). High Mobility Group Box 1 Protein: Possible Pathogenic Link to Atrial Fibrillation. Chin. Med. J..

[88] Qu C., Wang X.-W., Huang C., Qiu F., Xiang X.-Y., Lu Z.-Q. (2015). High Mobility Group Box 1 Gene Polymorphism Is Associated with the Risk of Postoperative Atrial Fibrillation after Coronary Artery Bypass Surgery. J. Cardiothorac. Surg..

[89] Li L., Liu Y., Li X., Qiu C. (2020). Insights into the Genetic Basis of HMGB1 in Atrial Fibrillation in a Chinese Han Population. Cardiovasc. Diagn Ther..

[90] Biscetti F., Rando M.M., Nardella E., Cecchini A.L., Pecorini G., Landolfi R., Flex A. (2019). Molecular Sciences High Mobility Group Box-1 and Diabetes Mellitus Complications: State of the Art and Future Perspectives. Int. J. Mol. Sci..

[91] Gunasekaran M.K., Viranaicken W., Girard A.-C., Festy F., Cesari M., Roche R., Hoareau L. (2013). Inflammation Triggers High Mobility Group Box 1 (HMGB1) Secretion in Adipose Tissue, a Potential Link to Obesity. Cytokine.

[92] Lumeng C.N. (2013). Innate Immune Activation in Obesity. Mol. Asp. Med..

[93] Feinkohl I., Winterer G., Pischon T. (2016). Obesity and Post-Operative Cognitive Dysfunction: A Systematic Review and Meta-Analysis. Diabetes Metab. Res. Rev..

[94] Hudetz J.A., Patterson K.M., Amole O., Riley A., Pagel P.S. (2011). Postoperative Cognitive Dysfunction after Noncardiac Surgery: Effects of Metabolic Syndrome. J. Anesth..

[95] Peppard P.E., Young T., Barnet J.H., Palta M., Hagen E.W., Hla K.M. (2013). Increased Prevalence of Sleep-Disordered Breathing in Adults. Am. J. Epidemiol..

[96] Lam E.W.K., Chung F., Wong J. (2017). Sleep-Disordered Breathing, Postoperative Delirium, and Cognitive Impairment. Anesth. Analg..

[97] Wagner S., Sutter L., Wagenblast F., Walther A., Schiff J.-H. (2021). Short Term Cognitive Function after Sevoflurane Anesthesia in Patients Suspect to Obstructive Sleep Apnea Syndrome: An Observational Study. BMC Anesthesiol..

[98] Wu K.-M., Lin C.-C., Chiu C.-H., Liaw S.-F. (2010). Effect of Treatment by Nasal Continuous Positive Airway Pressure on Serum High Mobility Group Box-1 Protein in Obstructive Sleep Apnea. Chest.

[99] Liu X., Ma Y., Ouyang R., Zeng Z., Zhan Z., Lu H., Cui Y., Dai Z., Luo L., He C. (2020). The Relationship between Inflammation and Neurocognitive Dysfunction in Obstructive Sleep Apnea Syndrome. J. Neuroinflamm..

[100] Min H.J., Park J.S., Kim K.S., Kang M., Seo J.H., Yoon J.H., Kim C.H., Cho H.J. (2021). Serum High-Mobility Group Box 1 Protein Level Correlates with the Lowest SaO2 in Patients with Sleep Apnea: A Preliminary Study. Braz. J. Otorhinolaryngol..

[101] Benítez-Burraco A., Grabrucker A.M., Dinan T.G., Kelly J.R., Minuto C., Cryan J.F., Clarke G. (2017). Cross Talk: The Microbiota and Neurodevelopmental Disorders. Front. Neurosci..

[102] Tang W., Meng Z., Li N., Liu Y., Li L., Chen D., Yang Y., Ling Z., Santos A. (2021). Roles of Gut Microbiota in the Regulation of Hippocampal Plasticity, Inflammation, and Hippocampus-Dependent Behaviors. Front. Cell Infect. Microbiol..

[103] Xu X., Hu Y., Yan E., Zhan G., Liu C., Yang C. (2020). Perioperative Neurocognitive Dysfunction: Thinking from the Gut?. Aging.

[104] Zhan G., Hua D., Huang N., Wang Y., Li S., Zhou Z., Yang N., Jiang R., Zhu B., Yang L. (2019). Anesthesia and Surgery Induce Cognitive Dysfunction in Elderly Male Mice: The Role of Gut Microbiota. Aging.

[105] Meng F., Li N., Li D., Song B., Li L. (2019). The Presence of Elevated Circulating Trimethylamine N-Oxide Exaggerates Postoperative Cognitive Dysfunction in Aged Rats. Behav. Brain Res..

[106] Singh G.B., Zhang Y., Boini K.M., Koka S. (2019). High Mobility Group Box 1 Mediates TMAO-Induced Endothelial Dysfunction. Int. J. Mol. Sci..

[107] Kant I.M.J., de Bresser J., van Montfort S.J.T., Slooter A.J.C., Hendrikse J. (2017). MRI Markers of Neurodegenerative and Neurovascular Changes in Relation to Postoperative Delirium and Postoperative Cognitive Decline. Am. J. Geriatr. Psychiatry.

[108] Nishibori M., Wang D., Ousaka D., Wake H. (2020). High Mobility Group Box-1 and Blood-Brain Barrier Disruption. Cells.

[109] Paudel Y.N., Angelopoulou E., Piperi C., Othman I., Shaikh M.F. (2020). Implication of HMGB1 Signaling Pathways in Amyotrophic Lateral Sclerosis (ALS): From Molecular Mechanisms to Pre-Clinical Results. Pharmacol. Res..

[110] Paudel Y.N., Angelopoulou E., Piperi C., Othman I., Aamir K., Farooq Shaikh M. (2020). Impact of HMGB1, RAGE, and TLR4 in Alzheimer’s Disease (AD): From Risk Factors to Therapeutic Targeting. Cells.

[111] Kang R., Chen R., Zhang Q., Hou W., Wu S., Cao L., Huang J., Yu Y., Fan X.G., Yan Z. (1998). HMGB1 in Health and Disease. Mol. Asp. Med..

[112] Ojeda B., Dueñas M., Salazar A., Antonio Mico J., Miguel Torres L., Failde I. (2018). Factors Influencing Cognitive Impairment in Neuropathic and Musculoskeletal Pain and Fibromyalgia. Pain Med..

[113] Otoshi K., Kikuchi S., Kato K., Sekiguchi M., Konno S. (2011). Anti-HMGB1 Neutralization Antibody Improves Pain-Related Behavior Induced by Application of Autologous Nucleus Pulposus onto Nerve Roots in Rats. Spine.

